# Economic impact of spectral body imaging in diagnosis of patients suspected for occult cancer

**DOI:** 10.1186/s13244-021-01116-0

**Published:** 2021-12-20

**Authors:** Michael Brun Andersen, Dyveke Ebbesen, Jesper Thygesen, Matthijs Kruis, Qing Gu, Ekta Dharaiya, Finn Rasmussen

**Affiliations:** 1 Department of Radiology, Copenhagen University Hospital, Gentofte Hospitalsvej 1, 2900 Hellerup, Denmark; 2 Department of Radiology, Zealand University Hospital, Roskilde, Denmark; 3 Department of Radiology, Aarhus University Hospital, Skejby, Denmark; 4 Central Denmark Region, Department of Clinical Engineering, Aarhus University Hospital, Skejby, Denmark; 5 Philips Healthcare, Best, The Netherlands; 6 Philips Healthcare, Cleveland, USA

**Keywords:** Spectral CT, Contrast enhanced CT, Healthcare economics

## Abstract

**Background:**

Based on prior studies spectral CT has shown a higher sensitivity for malignant lesions than conventional CT at the cost of lower specificity. For the radiologists, it also offers a higher degree of certainty in the diagnosis of benign lesions. The objective of this study was to evaluate the economic impact of spectral CT in patients suspected of occult cancer in a medical center in Denmark.

**Methods:**

This study was a secondary analysis using de-identified data from a prospective study of patients receiving a contrast-enhanced spectral CT scan. Based on suggested follow-up examinations on both spectral CT and contrast-enhanced CT, costs from a payer’s perspective were determined using unit costs obtained from national databases.

**Results:**

The dataset contained 400 patients. Overall, 203 follow-up procedures were eliminated based on spectral data reading. The largest reduction in suggested follow-up procedures was found for the kidney (83%), followed by the liver (66%), adrenal glands (60%), and pancreas (42%). The total estimated costs for suggested follow-up procedures based on spectral data reading were €155,219, 25.2% (€52,384) less than that of conventional CT reading.

**Conclusion:**

Our results provide support for spectral body imaging as an advanced imaging modality for suspected occult cancer. A substantial number of follow-up diagnostic procedures could be eliminated based on spectral data reading, which would result in significant cost savings.

**Supplementary Information:**

The online version contains supplementary material available at 10.1186/s13244-021-01116-0.

## Key points

- A total of 203 follow-up procedures were eliminated based on spectral data.
- The largest reduction in suggested follow-up procedures was found for the kidney (83%), followed by the liver (66%), adrenal (60%), and pancreas (42%).
- The total estimated cost for suggested follow-up procedures based on spectral data reading was €155,219, 25.2% (€52,384) less than that of conventional CT reading.

## Background

Cancer prevalence and mortality rates in Denmark are among the highest in Europe [1–3]. Diagnosing cancer is challenging, especially in patients with vague symptoms, who have a longer time to diagnosis and a higher mortality compared to those presenting with classical symptoms of malignant disease [4, 5]. In 2009, fast-track investigational courses for patients with suspected cancer were introduced in Denmark to streamline organ-specific cancer patient pathways (CPPs). Several CPP iterations have been made, and in 2012, a new CPP for patients with non-specific symptoms of serious illness (NS-CPP) that could be cancer was implemented. The goal was an accelerated investigational course of no longer than 9 days to ensure relevant triage into either an organ-specific CPP or referral to relevant specialties to take care of treatment based on the diagnostic findings made during the workup [4–7].

Patients presenting with non-specific symptoms (e.g., fatigue, pain, fever, unintended weight loss, abnormal blood chemistry) were initially screened for occult malignancy with a physical examination and laboratory evaluation performed by the general practitioner. In case of continued suspicion, initial screening was supplemented with diagnostic contrast-enhanced computed tomography (CE-CT) of the thorax, abdomen, and pelvis [4, 5, 8].

Several techniques have been investigated to increase the efficacy of the diagnostic workup. A randomized prospective trial investigating the clinical performance of ^18^F-FDG PET/CT showed that ^18^F-FDG PET/CT has a higher diagnostic specificity and accuracy for detecting cancer in patients with suspected serious illness compared to conventional CT. The author also pointed out that ^18^F-FDG PET/CT could save expensive additional procedures as well as secondary ^18^F-FDG PET/CT scans. However, this potential cost savings have not been assessed [9].

Contrast-enhanced spectral CT (CE-SCT) is a novel technology that uses two layers of detectors to simultaneously collect low-and high-energy data. A recent study comparing the effectiveness of CE-SCT to contrast-enhanced CT (CE-CT) in a prospectively enrolled patient cohort that entered the NS-CPP found that CE-SCT detected more lesions than CE-CT, but with a slightly lower specificity. However, CE-SCT increased the radiologist’s confidence in the correct characterization of various lesions [10].

The Danish reimbursement system for radiological procedures is a mixed system. It is a combination of block hospital budgets and activity targets. The first part is the set maximum cap for the department’s budget. The second part is a diagnosis-related grouping (DRG) system, in which all diagnoses and procedures are coded. Each code represents a monetary value paid to the department responsible for the treatment or procedure. We can use the activity-based part of the system to determine the cost of individual diagnostic procedures in the Danish healthcare system.

The purpose of this study was to perform an economic analysis of the cost of downstream diagnostic procedures following CE-SCT compared to CE-CT in patients suspected of having occult cancer in the Danish healthcare system. Specifically, we hypothesized that the direct cost of follow-up diagnostic procedures would be less based on CE-SCT reading compared to CE-CT reading during occult cancer diagnostic workups.

## Methods

### Study design and setting

This was a secondary analysis of a cohort of prospectively enrolled patients with suspected serious illnesses that could be cancer. Study subjects were included between May 2017 and November 2018. The ethical committee waived ethical issues in cases where all participants provided written consent. This study was approved by the Danish Data Authority.

### Patients

**Fig. 1:**
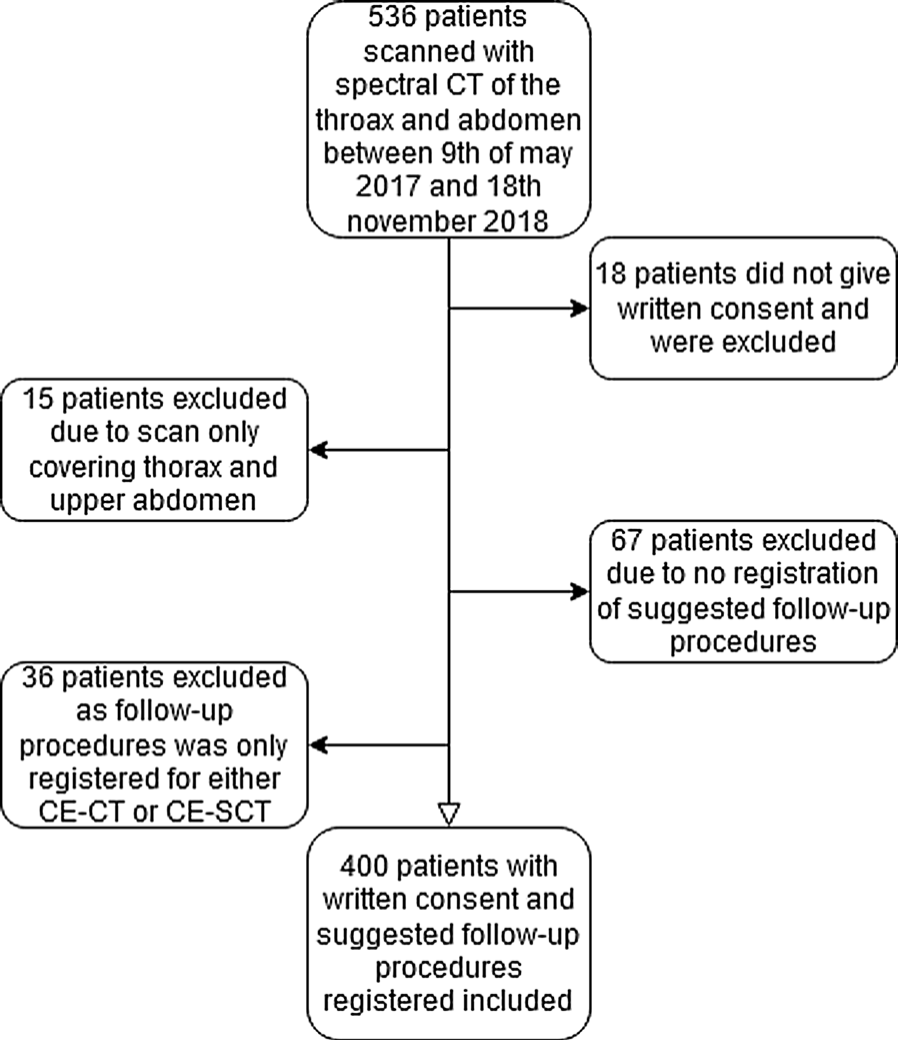
Flowchart of inclusion and exclusion

A total of 536 patients, referred by their general practitioner (GP) to the NS-CPP, were prospectively enrolled during the study period. A total of 503 patients provided written consent to participate in the project and allowed access to images and clinical data. Patients referred from the GP to the NS-CPP were eligible for inclusion. Exclusion criteria were missing written consent, scan protocol differing from the national guidelines, and allergies to iodine contrast media. In this analysis, a subpopulation of 400 patients was used, where the radiologists recorded follow-up or supplemental examinations (Fig. 1). The demographics of the patient population are shown in Table 1.

**Table 1:** Patient demographics: gender, age, weight, height, and radiation dosage

	*n*	Mean	SD	95% confidence interval	Min	Max
Males	173					
Females	227					
Age (yh)	400	64.42	13.00	63.14–65.70	19	90
Height (cm)	393	171.63	8.98	170.73–172.52	147	196
Weight (kg)	395	73.84	15.32	72.32–75.35	33	150
Dose Length Product (mGy × cm)	400	2112.33	432.35	2069.83–2154.83	1081.1	4778

### Spectral contrast enhanced CT and contrast enhanced CT

SCE-CT of the chest, abdomen, and pelvis were acquired using a 64-row dual-layer detector CT scanner (Philips IQon; Philips Healthcare). CT acquisition parameters were 64 × 0.625 mm collimation, kVp 120–140, mAs/slice 150–250, rotation time 0.75 s, reconstruction thickness 2 mm, increment 1 mm, pitch 1.078, FOV 35 cm and matrix 512 × 512. Iodixanol 270 mg/mL (Visipaque® 270; GE Healthcare), or iohexol 300 mg/mL (Omnipaque® 300; GE Healthcare), was injected intravenously in weight-adjusted doses of 2 mL/kg body weight to compensate for differences in distribution volume, with an injection rate of 4 mL/s. A bolus tracking technique was used with an ROI in the descending aorta at the level of Carina to compensate for differences in cardiac output. A threshold of 150 HU was used, and CT was performed after a delay of 15 s for the chest and upper abdomen (late arterial phase), and 65 s for the abdomen (portal venous phase). The mean dose length product (DLP) of CT scans performed on the population was 2104 mGy × cm (CI95% 2064–2144). By spectral separation of the CT signal in the two detector layers, a spectral CT dataset was reconstructed. By weighted addition of the signal of the two layers before reconstruction, a conventional CT dataset was reconstructed, which possesses all the features of a normal single energy CT in terms of dose and image quality.

### Reading of examinations and determination of follow-up procedures

Scan data were transferred to a dedicated workstation for spectral CT image interpretation (Intellispace 9; Philips Healthcare) and divided into primary and secondary reading folders. The reading folders either contained the CE-CT or a superset of the conventional results and CE-SCT. The spectral data included virtual monoenergetic images (ranging from 40 to 200 keV), effective atomic number (Z_eff_, reporting the atomic number of the tissue), and iodine-no-water (pure spectral decomposition of iodine and water. Calcium remained visible and was mimicked by iodine), iodine density (similar to iodine no water, but calcium was masked out), contrast enhancing structures (masking of iodinated tissues), uric acid (masking of uric acid containing tissue), and virtual non-contrast (VNC, a 70 keV map without the signal of iodine contrast).

Overlay images on the conventional series or the VNC were available for all datasets.

The readings were performed in consensus by two radiologists with 9 and 33 years of experience. A minimum of 3 months between reading the CE-CT or CE-SCT from the same patient was maintained to eliminate recall bias. All findings were entered into the RedCAP database [11, 12]. The readers had access to all spectral information. The primary assessment was performed on the virtual low monoenergetic series (between 40 and 50 keV). Lesions identified in the low monoenergetic series were subsequently characterized using the remaining spectral series (iodine density, z-effective, and virtual non-contrast). The reading time for each examination was recorded using a stopwatch. The time was started after the images had been loaded onto the workstation and when an assessment of the images was initiated. The time was stopped when the examination was concluded. Further details regarding the reading of the individual series are presented earlier [10].

For the 400 patients, the readers recorded the need for follow-up examinations based on best clinical practice and international guidelines [13–16]. This covered a wide range of examinations, including CT thorax in cases of pulmonary nodules according to the Fleischner Society criteria, multiphase CT or ultrasound (US) in case of hyperdense lesions in the kidney, PET/CT in cases of suspected lung cancer, transvaginal US for ovarian lesions, dedicated adrenal CT for adrenal lesions, US, and possible biopsy from thyroid lesions.

### Outcomes

For the present analysis, the primary outcome was the reduction in suggested follow-up examinations and the estimated overall direct cost savings for the whole cohort. The secondary outcome was the reduction of suggested follow-up examinations and estimated direct cost savings for the subgroup of patients with suspected prostate, liver, adrenal, kidney, pancreas, and lung lesions.

### Cost estimation

An economic analysis was performed from the Danish payer perspective. All cost estimates are presented in the 2020 Euro.

As the parent study did not collect cost data, we obtained the unit cost from the national data source [10, 17]. Direct costs were calculated for each patient for any follow-up procedures suggested for all organ systems. The original costs were derived in Danish Krone and then converted to euro. Furthermore, the economic costs in accordance with the U.S. system were also calculated to show the impact of the results on health care systems with other reimbursement structures [18]. The unit cost of the follow-up procedures in the U.S. was estimated using the national average payment allowance by US Centers for Medicare and Medicaid Services Medicare Physician Fee Schedule and Hospital Outpatient Prospective Payment by Current Procedural Terminology (CPT) codes [19]. All US cost estimates are presented in US dollars by 2020 (Additional file 1).

## Results

A total of 573 follow-up procedures were suggested for CE-CT and 370 for CE-SCT. A total reduction of 203 follow-up procedures was achieved using CE-SCT. Fifty-eight (83%) follow-up procedures were eliminated for the kidney, which was the largest reduction among all organs. For liver, adrenal, and pancreas, the number of reduced examinations were 78(66%), 25(60%), and 5(42%) respectively. The number of reductions in the suggested follow-up procedures was less than 10 (10%) for the prostate and lung (Table 2).

**Table 2:** Direct cost analysis in Denmark and US by organs, CE-CT compared to CE-SCT

Area of interest	Frequency of follow-up following CE-CT	Frequency of follow-up following CE-SCT	Overall reduction in follow-up procedures	Healthcare system	Cost of follow-up after CE-CT	Cost of follow-up after CE-SCT	Reduction in costs	Reduction in percent (%)
Over all	573	370	203	DK	€ 207.603	€155.219	€ 52.384	25.2
				USA	$376.891	$3.8.054	$68.837	18.2
Liver	118	40	78	DK	€ 37.263	€18.796	€ 18.467	49.6
				USA	$81.155	$44.341	$36.814	45.4
Kidney	70	12	58	DK	€ 18.041	€3.967	€ 14.074	78.0
				USA	$23.697	$16.989	$6.708	28.3
Adrenal	42	17	25	DK	€ 11.465	€4.367	€ 7.098	61.9
				USA	$20.007	$7.621	$12.386	61.9
Pancreas	12	7	5	DK	€ 7.736	€6.066	€ 1.670	21.6
				USA	$11.858	$8.876	$2.982	25.2
Lung	78	72	6	DK	€ 40.625	€42.418	€ − 1.793	− 4.4
				USA	$52.205	$51.725	$480	0.9
Prostate	59	54	5	DK	€ 3.021	€3.545	€ − 524	− 17.4
				USA	$10.279	$20.074	− $9.795	− 95.3

In most organ systems, we found reductions in overall costs of follow-up procedures, except for prostate and lungs, where we saw an increase in costs, although the reduction in the number of suggested follow-up examinations was consistent with the rest of the organ systems (Table 2).

Even though there were differences in unit costs of diagnostic procedures between the Danish and U.S. healthcare systems, when we compared savings in percentages, the same trends became visible. The largest differences were observed in the kidney and prostate. In the Danish system, we found a reduction of 79.01% in the kidney, where we only found a reduction of 28.3% in the US system. For the prostate, we found an increase in costs of 17.4% in the Danish system and an increase of 95.3% in the U.S. system.

From the Danish payer perspective, the total estimated costs of the follow-up procedures based on CE-SCT were €155,219, 25.2% (€52,384) less than that of CE-CT. The estimated costs per patient based on CE-SCT were €371, while the estimated costs based on CE-CT readings were 35% higher (€501), and the estimated total cost savings per patient for the follow-up examinations were €130.

A breakdown of the frequency of suggested follow-up procedures is presented in Table 3. The unit costs for individual procedures can be found in the Additional file 1.

**Table 3:** Overview of recommended follow-up procedures by organs, CE-CT compared to CE-SCT

Area of interest	Scan type	Frequency	Suggested procedure
Liver	CE-CT	8	Biopsy of the liver
		1	Endoscopic US of the liver
		3	Endoscopic retrograde cholangio pancreaticography
		2	Gastroscopy
		4	Magnetic resonance cholangio pancreaticography
		5	MRI scan of the liver
		1	18F-FDG PET/CT
		94	US examination of the liver
	CE-SCT	4	Biopsy of the liver
		4	Endoscopic US of the liver
		3	Endoscopic retrograde cholangio pancreaticography
		3	Magnetic resonance cholangio pancreaticography
		5	MRI scan of the liver
		21	US examination of the liver
Kidney	CE-CT	4	Biopsy of the kidney
		1	CT scan of the kidney
		1	MRI of the kidney
		64	US examination of the kidney
	CE-SCT	8	Biopsy of the kidney
		1	CT scan of the kidney
		3	US examination of the kidney
Adrenal	CE-CT	42	Dedicated CT of the adrenal gland
	CE-SCT	1	Biopsy of the adrenal
		16	Dedicated CT of the adrenal gland
Pancreas	CE-CT	1	Biopsy of the pancreas
		3	Dedicated CT of the pancreas
		3	Endoscopic US of the pancreas
		4	MRI of the pancreas
		1	18F-FDG PET/CT
	CE-SCT	1	Biopsy of the pancreas
		2	Dedicated CT of the pancreas
		4	Endoscopic US of the pancreas
Lung	CE-CT	1	Biopsy of the lung
		57	CT scan of the lungs
		20	18F-FDG PET/CT
	CE-SCT	49	CT scan of the lungs
		3	Flexible bronchoskopy
		20	18F-FDG PET/CT
Prostate	CE-CT	4	Biopsy of the prostate
		53	PSA measurement
		1	MRI scan of the prostate
		1	US examination of the prostate
	CE-SCT	9	Biopsy of the prostate
		45	PSA measurement

## Discussion

Since the first clinical CT scanner capable of dual-energy CT was introduced in 2008, several studies of spectral/dual energy CT have shown numerous clinical benefits; however, very few have looked at the economic benefits of the technique [20, 21].

In this study, we used a scanner with a dual-layer detector capable of performing spectral acquisitions without the need to select the option prior to the examination. This made it possible to read both the CE-CT and CE-SCT datasets. Reporting the need for follow-up procedures on a per-lesion basis, we found a significant reduction in overall costs.

A retrospective study of 2401 patients reported that dual-energy CT-generated iodine maps, when used as part of routine radiology workflow, were associated with lower rates of recommendations for additional radiologic studies compared to conventional CT scans due to incomplete diagnosis or characterization (9.1% vs. 11.9%, *p* = 0.01). However, cost savings have not been directly measured [21]. In a somewhat comparable study, Itani et al. retrospectively investigated the cost associated with follow-up imaging procedures in 69 patients presenting with abdominal symptoms. All patients underwent single-phase contrast-enhanced dual-energy CT, which characterized 27 incidental findings and accounted for cost savings of 15 additional imaging examinations [19]. They reported a total reduction in cost per patient of $85 compared to €130 ($154) found in the study presented here. One plausible reason was that in the current study, all follow-up procedures based on primary recommendations were included, and only follow-up procedures involving imaging modalities were included in previous studies.

**Fig. 2:**
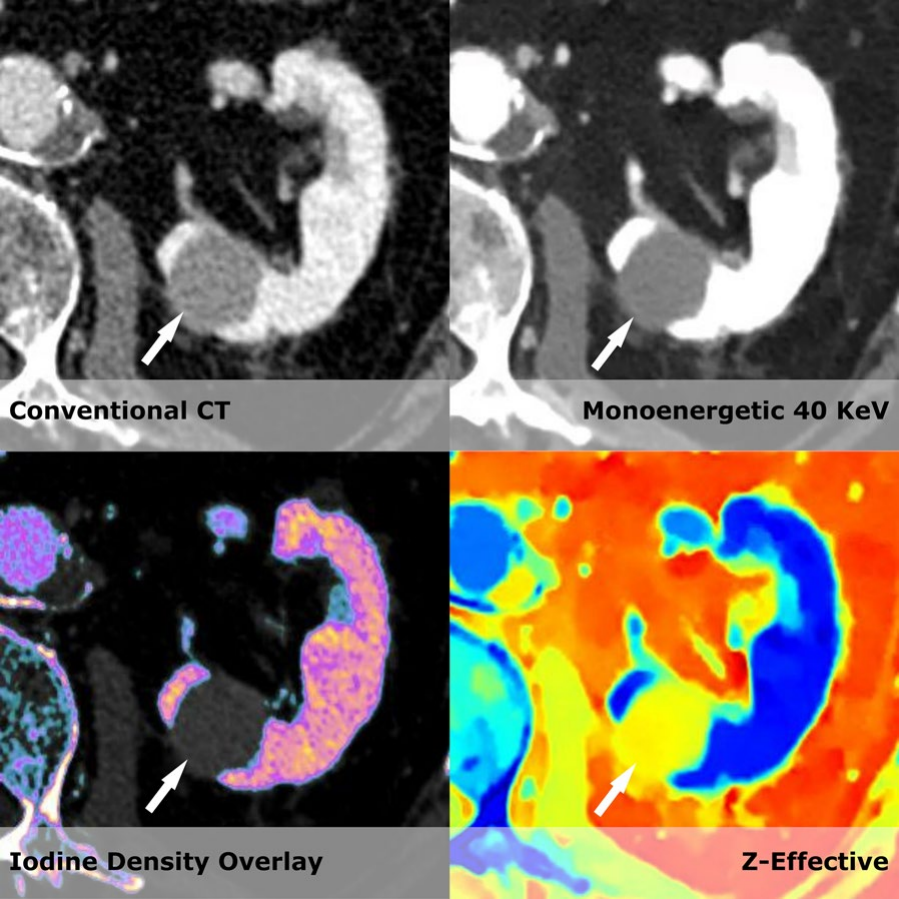
Shows a hyperdense lesion (79 HU on conventional CT images). However, it becomes clear when the spectral series are used that it is in fact a hyperdense cyst within the kidney that would be classified as a Bosniak type II as it is less than 3 cm and do not enhance (Iodine density 0.01 mg/mL, 40 monoenergetic with HU values of 83). To further increase the confidence of the reader the z-effective values are 7.24 corresponding to water. US later confirmed the diagnosis of a hyperdense cyst

**Fig. 3:**
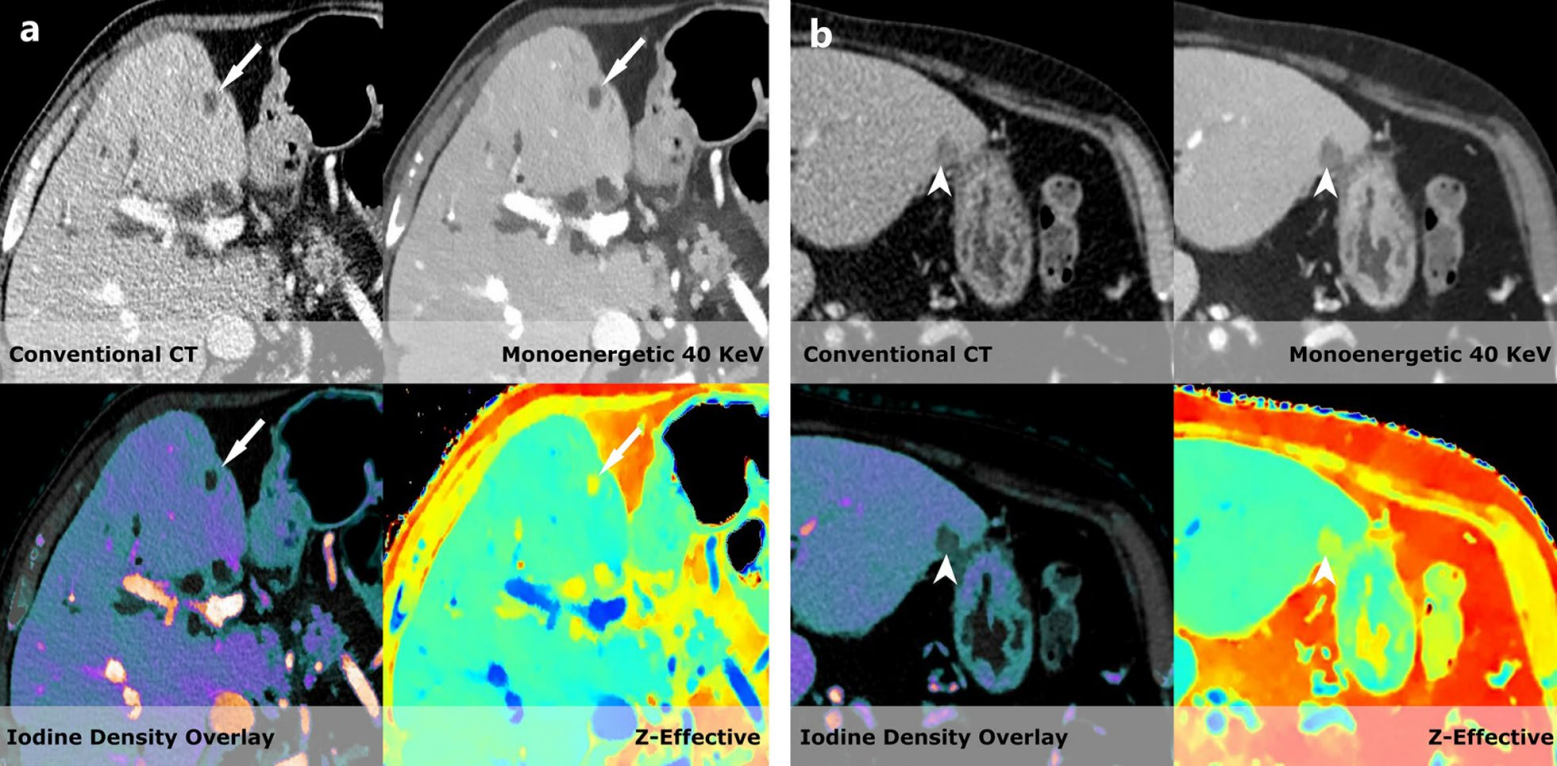
**a** In a patient with proven pancreatic cancer a hypodense lesion (white arrow) is seen in the right lobe of the liver. It shows HU values of 26 and has unsharp edges on the conventional scan. On Z-effective values of 7.39 are seen suggesting water content and there is no iodine uptake found on the iodine overlay (0.1 mg/mL which is below the detection rate). **b** In a patient with proven lung cancer a lesion (white arrowhead) similar to the one shown in **a** with HU values of 47 on the conventional series. On the spectral series; however, we find an increase to 84 HU on the low virtual monoenergetic scan suggesting iodine enhancement. This is proven on the iodine density with a measurement of 0.56 mg/mL. It was later proven by biopsy to be a liver metastasis from the primary lung cancer

**Fig. 4:**
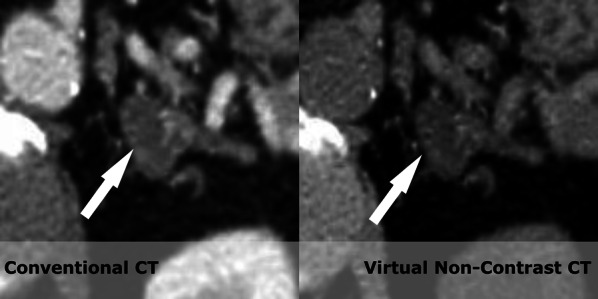
A lesion (white arrow) in the left adrenal is seen with HU values of 46 on the conventional portal venous phase scan. On the virtual non contrast series HU value of 2 was measured. As no malignant findings were made in the patient the correct diagnosis of an adenoma was made. This was confirmed by a true non contrast CT examination

In the liver, kidney, adrenal gland, and pancreas, we found a substantial reduction in estimated costs of suggested follow-up examinations; however, for the lung and prostate, the estimated costs increased in the CE-SCT group. In the liver and kidney, the major driver of the reduction in costs was a decrease in the need for the supplementary US to resolve the nature of suspected cystic lesions (Figs. 2 and 3a and b). For the adrenal gland, the estimated cost savings were associated with a decrease in the need for supplementary dedicated adrenal gland CT from 42 to 17. This was because spectral CT could characterize the lesion as an adenoma using virtual non-contrast scans (Fig. 4).

In the prostate, the estimated costs of follow-up procedures increased, even though a reduction in suggested follow-up examinations was observed (from 59 to 54). The surge in both countries was driven by a large unit cost of biopsies, where 10 were suggested following CE-SCT reading compared to the 4 suggested following CE-CT reading. PSA measurements were suggested for the six cases in which biopsies were not suggested in the first place following CE-CT reading. If elevated PSA (> 4 ng/mL) levels were detected, they would have subsequently received a biopsy. Unfortunately, we did not perform PSA measurements for all patients with an enlarged prostate. However, most likely biopsies would have been performed at a later stage during the prostate lesion diagnosis workup following CE-CT. Therefore, CE-SCT would probably have accelerated the investigational course and most likely showed an overall decrease in costs for follow-up procedures in the prostate.

**Fig. 5:**
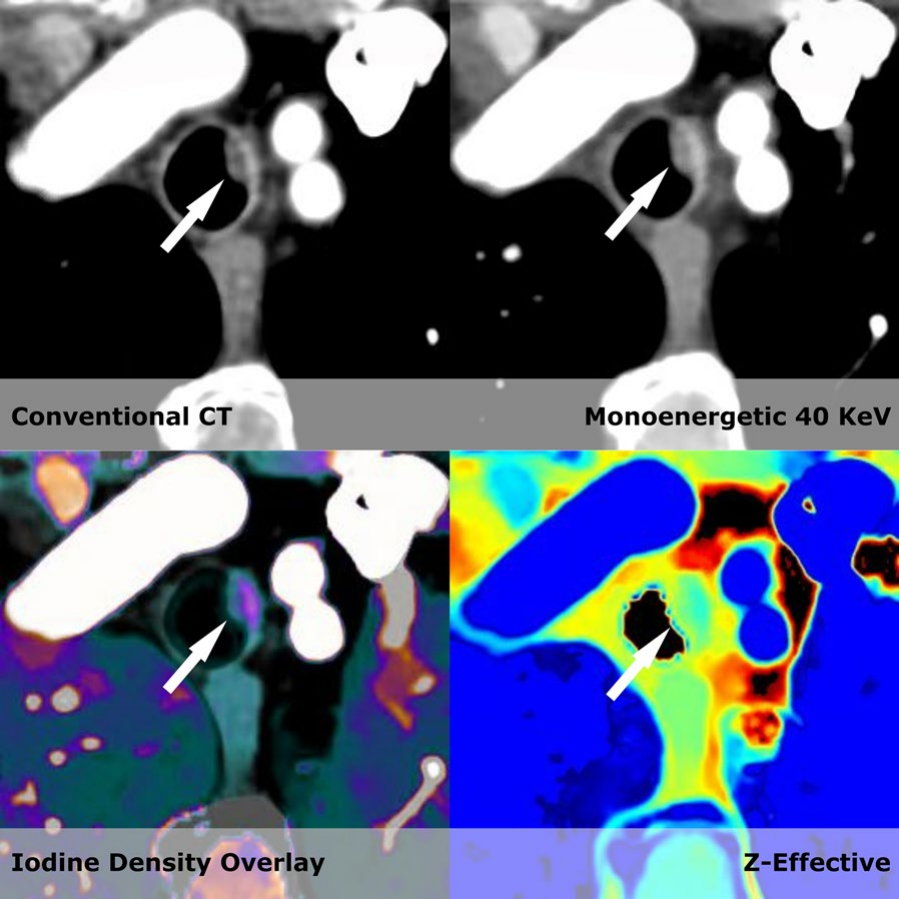
On the left side of the trachea a lesion traversing the wall with a soft-tissue component are seen (white arrow). However, the lesion could also represent intratracheal mucus. But during the reading of the CE-CT, it was missed. On the CE-SCT it was seen both on the virtual low monoenergetic and the Z-effective map which led to a suggestion of flexible bronchoscopy. Unfortunately, the patient was deceased by the time the CE-SCT was read and no definitive diagnoses were made

For the lungs, the overall number of suggested examinations reduced from 81 to 76; however, the total estimated costs slightly increased in the Danish system (4.4%). This is largely due to the fact that flexible bronchoscopy, a relatively expensive procedure in Denmark, was suggested for three patients to confirm lesions in the bronchi following CE-SCT reading, while none was suggested following CE-CT reading (Fig. 5). It is very likely if symptoms developed bronchoscopy would be performed during the further workup following CE-CT reading. In that case, CE-SCT can eliminate a step in the diagnostic process; however, our data cannot substantiate this.

It has been suggested that ^18^F-FDG PET/CT could be the first-line modality for patients suspected of having cancer. ^18^F-FDG PET/CT had a sensitivity of 83% and was not significantly different from CE-CT; however, it had higher specificity (96% vs. 85%; *p* = 0.028) and a higher accuracy [9]. Caspersen et al. reported that the sensitivity of ^18^F-FDG PET/CT for cancer detection in patients suspected of serious disease was 81.0%, but with a specificity of only 76.4% [22]. In a previously published clinical study, the sensitivity of CE-SCT for cancer findings was 89%, with a slightly lower specificity, mainly due to lesions found in the prostate that currently cannot be fully characterized using this technique [10]. As the patient populations were comparable in both studies (same country, same cancer package, same cancer incidence, same inclusion criteria), CE-SCT seemed to be on par with or even better than ^18^F-FDG PET/CT for the diagnosis of malignancy. From the payer perspective, the reimbursement rate of ^18^F-FDG PET/CT (€1234.57) was 4.5 times as high as that of the CE-SCT scan (€272.93) within the Danish system. From the provider’s perspective, the acquisition cost of a dual energy/spectral CT scanner was much lower than that of a PET/CT scanner. In addition, many hospitals did not have a nuclear medicine department; therefore, additional staff training and acquisition/access to a cyclotron would be required. Our results indicated that spectral CT had comparable diagnostic performance to F-FDG PET/CT and would bring more economic benefits to key stakeholders.

The time to diagnosis is crucial. In many countries, access to ^18^F-FDG PET/CT scanners, time from referral, cost of the examination, and access to tracers were significant barriers. The lower acquisition cost and better access to dual energy/spectral CT make it ideal as a first-line examination. Making correct diagnoses with the first examination would also reduce time to diagnosis as well as patient’s concern as an uncertain diagnosis that would require further workup.

There are several limitations to the current study. First, the cost estimation was purely based on clinical recommendations and not from recorded claims data. However, the decision rationale was carefully documented for clinical routine or diagnostic certainty and in consensus by two radiologists. Radiologists’ recommendations reflected the direct impact of CE-SCT on diagnostic confidence and clinical decision-making. Second, there were differences between patient demographics, disease conditions, and reimbursement systems worldwide, which can make direct comparisons challenging. Furthermore, there can be false negative results based on a misinterpretation of CE-SCT series; however, this can also be seen in CE-CT. Potentially this can deprive patients of needed follow-up examinations and has to be taken into account as the cost savings related to CE-SCT is indeed to minimize follow-up examinations. Finally, although not a limitation, we did note that the device and physician costs needed to be carefully assessed. The increased acquisition cost of a spectral CT system compared to a lower-end CT system can be a burden to the provider. Additional prospective studies following the entire care pathway are needed to assess the underlying economic impact, including time to diagnosis, time to treat, and overall health outcomes, to key stakeholders. The published clinical trial reported that, on average, the radiologist spent additional 82 s reading a CE-SCT compared to a CE-CT [10]. In both, Denmark and the US, radiologists were currently paid the same amount for both readings. Although not reflected in the physician fee schedule, the consumption of physician time needs to be considered.

In conclusion, CE-SCT decreases the need for follow-up examinations and reduces the overall costs for patients suspected of serious illnesses that could be cancer. The results suggest that the reduced costs associated with CE-SCT during occult cancer diagnosis workup could be considered as a potential benefit in decision making. These estimates provide evidence to inform the current practice over the use of CE-SCT from an economic standpoint to help guide the clinically appropriate use of this rapidly evolving technology.

## Supplementary Information

**Additional file 1**. Unit cost of procedures in Denmark; Unit cost of procedures in U.S.

## Data Availability

The data that support the findings of this study are not publicly available due to GDPR regulations. Data are however available from the authors upon reasonable request and only with the permission of the Danish data protection authorities and the Danish national ethical committee.

## Abbreviations

^18^F-FDG PET/CT: ^18^FluoroDeoxyGlucose positron emission tomography/computed tomography
CE-CT: Contrast enhanced computed tomography
CE-SCT: Contrast enhanced spectral computed tomography
CPP: Cancer patient pathway
DRG: Diagnosis related grouping
GP: General practitioner
NS-CPP: Non-specific cancer patient pathway
ROI: Region of interest
US: Ultrasound
