# Supplementary material for: Economic impact of spectral body imaging in diagnosis of patients suspected for occult cancer
Source: Insights Imaging. 2021 Dec 20;12:190. doi: 10.1186/s13244-021-01116-0 (PMC8688640; doi:10.1186/s13244-021-01116-0)
Supplement: Supplementary file 1 — Additional file 1. Unit cost of procedures in Denmark; Unit cost of procedures in U.S. [file 13244_2021_1116_MOESM1_ESM.docx]

**ELECTRONIC SUPPLEMENTARY MATERIAL**

**Supplemental material 1. Unit cost of procedures in Denmark**

| Procedure | Cost (€) |
| --- | --- |
| US examination of upper abdomen | 248.66 |
| CT scan of kidneys | 272.98 |
| Flexible bronchoscopy | 1450.27 |
| MRI scan of the entire body | 380.12 |
| MRI scan of spleen | 309.79 |
| MRI scan of genitalia feminine | 309.79 |
| MRCP | 309.79 |
| MRI scan of the upper abdomen | 380.12 |
| MRI scan of liver | 380.12 |
| MRI scan of the heart | 380.12 |
| MRI scan of pancreas | 380.12 |
| MRI scan of the mediastinum | 309.79 |
| MRI scan of prostate | 309.79 |
| Kidney MRI scanning | 380.12 |
| CT scan of adrenal glands | 272.98 |
| CT scan of lungs | 272.98 |
| endoscopic UL examination of liver | 1288.03 |
| US study of liver | 248.66 |
| Biopsy of liver | 368.51 |
| ERCP | 1288.03 |
| Lymph node exploration | 1059.79 |
| CT scan of pancreas | 272.98 |
| endoscopic UL examination of pancreas | 1288.03 |
| Biopsy of prostate | 259.56 |
| PSA measurement* | 26.87 |
| US examination of the bladder | 248.66 |
| US examination of prostate, including transrectal scanning | 248.66 |
| cystoscopy with biopsy | 883.35 |
| spleen biopsy | 368.51 |
| CT scan of the mediastinum | 272.98 |
| Diagnosis, laparotomy with systematic lymph node biopsies | 1032.66 |
| Laparoscopic internal gallbladder drainage | 707.07 |
| US study of genitalia feminine | 248.66 |
| Echocardiography | 245.44 |
| US examination of kidneys | 248.66 |
| US study of glandula thyroid and parathyroid | 248.66 |
| colonoscopy | 753.87 |
| Biopsy of pancreas | 368.51 |
| CT scan of the lower abdomen, incl. pelvis | 272.98 |
| Specific or unspecific functionally related to genital care and reproduction | 280.93 |
| Bronchoscopic US examination | 248.66 |
| Ebus | 248.66 |
| Gastroscopy | 707.07 |
| MRI scan of the lower abdomen, incl. pelvis | 309.79 |
| Biopsy of colon wall without colostomy | 373.62 |
| PET Tumor Scanning, F-18-FAZA | 1234.57 |
| Biopsy of lung | 373.62 |
| Esophagoscopy with biopsy | 404.3 |
| Gynecological investigation | 440.44 |
| Thyroid Scintigraphy, SPECT Diagnostic, I-131 Iodide | 407.03 |
| Mammography, screening | 89.33 |
| Transvaginal US examination of bowel | 248.66 |
| US examination of scrotum | 205.00 |
| HRCT of lungs | 260.52 |
| US examination of lower abdomen | 205.00 |
| Biopsy lymph nodes | 248.66 |
| Biopsy of kidney and renal pelvis | 368.51 |
| Arterial scleroses in lower extremity | 152.75 |
| clinical assessment of lymph nodes | 364.19 |
| urological assessment | 741.52 |
| examination of subcutaneous process clinically | 230.62 |
| Biopsy from the tumor in the upper abdominal muscles | 248.66 |
| Transvaginal US examination of genitalia feminine | 248.66 |
| Endoscopic US examination of esophagus | 1272.31 |
| Hematological investigation | 427.31 |
| needle biopsy of the mediastinum | 240.63 |
| cystoscopy mph fistula | 741.52 |

Source: Sundhedsdata-Styrelsen DRG takster 2020 (<http://interaktivdrg.sundhedsdata.dk/>). Original costs were derived in Danish Krone and then converted to Euro.

* Estimation provided by the clinician

**Supplemental material 2. Unit cost of procedures in U.S.**

| Procedure | Cost (USD) |
| --- | --- |
| US examination of upper abdomen | 236.12 |
| CT scan of kidneys | 434.18 |
| Flexible bronchoscopy | 1521.01 |
| MRI scan of the entire body | 667.39 |
| MRI scan of spleen | 791.32 |
| MRI scan of genitalia feminine | 505.18 |
| MRCP | 478.51 |
| MRI scan of the upper abdomen | 791.32 |
| MRI scan of liver | 791.32 |
| MRI scan of the heart | 560.68 |
| MRI scan of pancreas | 791.32 |
| MRI scan of the mediastinum | 960.7 |
| MRI scan of prostate | 439.24 |
| Kidney MRI scanning | 791.32 |
| CT scan of adrenal glands | 476.36 |
| CT scan of lungs | 401.04 |
| endoscopic US examination of liver | 1728.78 |
| US study of liver | 514.55 |
| Biopsy of liver | 1689.76 |
| ERCP | 4695.73 |
| Lymph node exploration | 3271.54 |
| CT scan of pancreas | 476.36 |
| endoscopic UL examination of pancreas | 1721.93 |
| Biopsy of prostate | 2128.25 |
| PSA measurement* | 20.44 |
| US examination of the bladder | 162.27 |
| US examination of prostate, including transrectal scanning | 242.97 |
| cystoscopy with biopsy | 2492.97 |
| spleen biopsy | 236.12 |
| CT scan of the mediastinum | 434.18 |
| Diagnosis, laparotomy with systematic lymph node biopsies | 1521.01 |
| Laparoscopic internal gallbladder drainage | 667.39 |
| US study of genitalia feminine | 791.32 |
| Echocardiography | 505.18 |
| US examination of kidneys | 478.51 |
| US study of glandula thyroid and parathyroid | 791.32 |
| colonoscopy | 791.32 |
| Biopsy of pancreas | 560.68 |
| CT scan of the lower abdomen, incl. pelvis | 791.32 |
| Specific or unspecific functionally related to genital care and reproduction | 960.7 |
| Bronchoscopic US examination | 439.24 |
| Ebus | 791.32 |
| Gastroscopy | 476.36 |
| MRI scan of the lower abdomen, incl. pelvis | 401.04 |
| Biopsy of colon wall without colostomy | 1728.78 |
| PET Tumor Scanning, F-18-FAZA | 514.55 |
| Biopsy of lung | 1689.76 |
| Esophagoscopy with biopsy | 4695.73 |
| Gynecological investigation | 3271.54 |
| Thyroid Scintigraphy, SPECT Diagnostic, I-131 Iodide | 553.15 |
| Mammography, screening | 138.39 |
| Transvaginal UL examination of bowel | 1251.89 |
| US examination of scrotum | 219.55 |
| HRCT of lungs | 401.04 |
| US examination of lower abdomen | 227.11 |
| Biopsy lymph nodes | 3270.1 |
| Biopsy of kidney and renal pelvis | 1984.2 |
| Arterial scleroses in lower extremity | 2016.39 |
| clinical assessment of lymph nodes | 3270.1 |
| urological assessment | 756.5 |
| examination of subcutaneous process clinically | 1557.86 |
| Biopsy from the tumor in the upper abdominal muscles | 1545.24 |
| Transvaginal US examination of genitalia feminine | 236.48 |
| Endoscopic US examination of esophagus | 1721.93 |
| Hematological investigation | 281.97 |
| Needle biopsy of the mediastinum | 1835.36 |
| cystoscopy mph fistula | 756.5 |

Source: CMS Physician Fee Schedule <https://www.cms.gov/Medicare/Medicare-Fee-for-Service-Payment/PhysicianFeeSched>. Accessed on 11 September 2020

CMS Hospital Outpatient Prospective Payment. https://www.cms.gov/Medicare/Medicare-Fee-for-Service-Payment/HospitalOutpatientPPS/Hospital-Outpatient-Regulations-and-Notices. Accessed on 11 September 2020
